# Hydrodeoxygenation of Bio-Derived Phenol to Cyclohexane Fuel Catalyzed by Bifunctional Mesoporous Organic–Inorganic Hybrids

**DOI:** 10.3389/fchem.2018.00216

**Published:** 2018-06-14

**Authors:** Liuye Mo, Wanjin Yu, Huangju Cai, Hui Lou, Xiaoming Zheng

**Affiliations:** ^1^Institute of Innovation & Application, Zhejiang Ocean University, Zhoushan, China; ^2^State Key Laboratory of Fluorinated Greenhouse Gases Replacement and Control Treatment, Zhejiang Research Institute of Chemical Industry, Hangzhou, China; ^3^Key Lab of Applied Chemistry of Zhejiang Province, Department of Chemistry, Zhejiang University, Hangzhou, China

**Keywords:** mesoporous silica, bifunctional catalyst, hydrodeoxygenation, bio-oil, phenol, cyclohexane

## Abstract

In this work, mesoporous silica materials SBA-15 functionalized with propyl/ phenyl-sulfonic acid group were synthesized and loaded with Pt to form bifunctional catalysts. SAXRD, WAXRD, N_2_ adsorption-desorption, TEM techniques were used to characterize the above bifunctional catalysts. These bifunctional catalysts were applied to the reaction of hydrodeoxygenation (HDO) of bio-derived phenol (PhOH) to produce cyclohexane fuel and showed very good catalytic performances. There were strong synergies between the metal sites and the acid sites on the bifunctional catalysts. This reaction of phenol HDO provides a model system for the catalytic upgrading of biomass-derived fuel.

## Introduction

Due to its abundance (15–30 wt % of wood-based biomass) and remarkably lower oxygen content than polysaccharides, lignin is a favorable feedstock for the production of biofuel, which is regarded as a promising energy alternative for fossil fuels (Huber et al., 2006). Currently, two-step processes are typical strategies to utilize lignin for biofuel production. In the first step, lignin is hydrolyzed (Kudsy and Kumazawa, 1999; Shabtai et al., 1999; Hepditch and Thring, 2000; Liu et al., 2008) or fast pyrolyzed (Meier et al., 1994; Thring and Breau, 1996; Britt et al., 2000; Dobele et al., 2007; Boateng et al., 2008; Ingram et al., 2008; French and Czernik, 2010) to depolymerize into a mixture of simple aromatic compounds (mostly phenols). Unfortunately, large quantities of reactive, unstable, and corrosive oxygenate compounds are contained in crude biofuel, which cannot be used directly as a vehicle fuel (Garcia-Perez et al., 2007). Therefore, the crude mixture from the first step must be upgraded into fuels in the second step. In order to simultaneously stabilize two main kind of reactive components, aldehydes and organic acids, in the bio-oil, we have newly developed a reaction system named one-step hydrogenation-esterification (OHE) reaction as a possible approach for biofuel upgrading (Tang et al., 2008; Yu et al., 2011). However, phenols with poor combustion performance could not be converted effectively during the OHE process (Yu et al., 2011). Therefore, the conversion of phenolic compounds into hydrocarbon fuels remains a challenge (Crossley et al., 2010). Hydrodeoxygenation (HDO) is regarded as a most attractive and effective method to convert phenolic compounds to alkanes for bio-oil upgrading (Huber et al., 2006). Traditional catalysts for HDO reaction were sulfide CoMo and NiMo/γ-Al_2_O_3_ catalysts, which are used in the hydrodesulfurization or hydrodenitrogenation process in petroleum refineries (Senol et al., 2005; Bui et al., 2009). However, it is well known that the fuels produced by sulfide catalysts will be contaminated as sulfur may be leached into the reaction liquids, and the catalysts will suffer from deactivation by water induction and coke accumulation (Yan et al., 2010). Lercher and Kou groups have reported a new highly efficient one-pot route for HDO of aqueous phenolic bio-oil to cycloalkanes over catalysts combining the noble catalysts with mineral acids or Brønsted acidic ionic liquids (Zhao et al., 2009, 2010; Yan et al., 2010). New progresses have been made over noble metal and transition metal catalysts combined with liquid acids or solid acids to upgrade biofuels via HDO of bio-derived phenolic compounds (Luska et al., 2015; Dongil et al., 2016; Lee et al., 2016). The good catalytic performance of the catalysts using mesoporous acidic solid materials as supports can be expected, as the bulky molecular of bio-derived phenolic compounds can easily access the active sites in the mesopores. Furthermore, the environmental benign solid acids can be recovered simply by filtration. Bifunctional catalysts of Pt/HY and Pt/HBeta were successfully used for phenolics HDO to produce hydrocarbons in fixed-bed configurations (Hong et al., 2010; Zhu et al., 2011). However, utilization of the bifunctional catalysts using mesoporous solid acid materials as supports for HDO of bio-derived phenolics was rarely reported. More recently, Xiao group has developed mesoporous zeolite ZSM-5 supported Ru to convert efficiently both small and bulky phenolic biomolecules via HDO to the corresponding alkanes owing to the open mesopores with abundant exposed acidic sites in the catalysts (Wang L. et al., 2015).

This paper focuses on the conversion of bio-derived phenol to cyclohexane via HDO reaction, a liquid fuel with good combustion properties, under mild reaction conditions over Pt bifunctional catalysts which possess functions of both hydrogenation and dehydration. The support of SBA-15 functionalized with organosulfonic acid was used, showing strong acidity and accessibility for bulky bio-derived molecules, may overcome the disadvantages both of the liquid acids and microporous solid acids.

## Experimental section

### Catalyst preparation

The catalysts used in this work were organic-inorganic hybrid SBA-15 materials, which were functionalized with propylsulfonic (arenesulfonic) acid groups and loaded with platinum. Organosulfonic acid-functionalized SBA-15 materials were synthesized as described elsewhere (Mbaraka and Shanks, 2006; Tang et al., 2010) with only slight modifications. Tetraethoxysilane (TEOS, 98%, Aldrich) and (3-mercaptopropyl)trimethoxysilane (MPTMS, 85 wt. %, Acros) or 2-(4-chloro-sulfonyl-phenyl) ethyl trimethoxysilane (CSPTMS, 50 wt. % in CH_2_Cl_2_, Acros) were adopted directly without further purification as the silica and the organosulfonic acid sources. Pluronic P123 (Aldrich), a triblock copolymer of polyethylene oxide-polypropylene oxide-polyethylene oxide with the molecular structure PEO_20_-PPO_70_-PEO_20_ (Mw = 5800), was used as a structure template to synthesizethe SBA-15. H_2_PtCl_6_ solution used as the platinum precursor was provided by Hangzhou Kaiming Catalyst Co., Ltd. Formaldehyde (HCHO, 37 wt.%, aqueous solution, Sinopharm Chemical reagents Co., China) was used as reducing agents. The functionalized SBA-15 hybrids with propylsulfonic or arenesulfonic acid groups were abbreviated as SBA-Pr and SBA-Ar, respectively. The nominal loading of platinum on the catalysts was fixed at 1 wt%.

#### Synthesis of 1%Pt/SBA-Pr and 1%Pt/SBA-Ar

In a typical synthesis of SBA-Pr, Pluronic P123 (4.00 g, 0.69 mmol) was dissolved in HCl solution (1.9 M, 125 mL) at room temperature. Then, keeping stirring the solution was heated to 40°C before the addition of TEOS (8.23 mL, 36.74 mmol). Before the addition of MTPMS (3.674 mmol) and H_2_O_2_ (12.86 mmol), ~45 min was allowed for prehydrolysis of TEOS. The resulting mixture was agitated for 24 h at 40°C and then aged for another 24 h at 110°C under static conditions. After that, the resulting solids were separated by filteration, washed with D.I. water and dried at 100°C in an oven for 8 h. The template in the as-synthesis samples was extracted by 10% v/v HCl in ethanol and refluxing for 36 h. To ensure complete removal of the template, fresh ethanol would be introduced after every 12 h.

Pt was loaded on SBA-Pr by reduction-deposition method. 1.00 g SBA-Pr was added into the aqueous solution containing 30.0 mL of D.I. water and 0.021 g of H_2_PtCl_6_. The Formaldehyde solution (10 mL) was used as reducing agent and added dropwise into the above aqueous suspension with vigorous stirring () at 60°C. The suspension was stirred for 24 h at 60°C to allow the reduction of Pt^4+^ to Pt^0^. After that, the suspension was filtered at room temperature and thoroughly washed with D.I. water until free of Cl^−^ in the filtrate (tested with silver nitrate solution). The wet filter cake was re-suspended in 50 mL of 1 wt% H_2_SO_4_ solution for 4 h for acidification. Finally, the solid was treated by filtering, washing, and vacuum drying at 110°C. The synthesis procedures of SBA-Ar and **1%Pt/SBA-Ar were** the same as the synthesis of SBA-Pr and 1%Pt/SBA-Pr described above, besides CSPTMS was used instead of MPTMS.

### Catalyst characterization

Small angle XRD (SAXRD) patterns were recorded for SBA-Pr, SBA-Ar, 1%Pt/SBA-Pr, and 1%Pt/SBA-Ar powder in order to confirm the hexagonal structure of samples. Wide angle XRD (WAXRD) was used to analyze the 1%Pt/SBA-Pr and 1%Pt/SBA-Ar. The above XRD measurements were performed on the Rigaku D/MAX-RB.

A Philips-FEI transmission electron microscope (Tecnai G2 F30 S-Twin, The Netherlands), operating at 300- kV, was used to obtain the HRTEM images of catalysts. Samples were mounted on copper grid-supported carbon films by dropping a few droplets of ultrasonically dispersed suspensions of samples in ethanol on the grids, followed by drying at ambient conditions.

CO chemisorption measurements were carried out at 25°C on a CHEMBET-3000 pulse flow system. Prior to measurements, the catalysts were tableted formed, crushed and sieved to 40–60 mesh. To remove any oxygen from the samples, pretreatments were done at 250°C for 1 h with a ramp of 10°C/min from room temperature under 30 mL/min of 5 vol. % H_2_/He up to 250°C. After that, the adsorbed hydrogen on samples was removed by purging with 30 mL/min helium gas for 2 h at 250°C. Finally, the catalysts were cooled down to 25°C under He flow and 5 vol% CO in helium were pulsed into the catalysts until the CO peak areas appeared to be identical.

The real weight percentages of Pt on the supports of SBA-Pr and SBA-Ar were determined by inductively coupled plasma-mass spectroscopy (ICP-MS) on a PS1000 instrument from American LEEMAN LABS INC.

The N_2_ adsorption/desorption method was adopted to measure the textural properties of the catalytic materials on an automated adsorption apparatus (OMNIISORP 100CX) at−196°C. Prior to the adsorption/desorption measurement, all samples were degassed for 2 h at 195°C in the pretreated chamber of the adsorption apparatus. The surface area, pore volume (Vp) were calculated by the Brunauer-Emmett-Teller (BET) and Barret-Joyner-Hallenda (BJH) methods. The mean pore diameter (MPD) of the samples was analyzed by the BJH method based on the adsorption branch of the N_2_ adsorption-desorption isotherms.

The decomposition of organic moiety and the thermal stability of the solids were examined by thermogravimetric analysis (TGA) on a Perkin-Elmer TGA7 instrument. The samples were heated under a stream of air (20 mL/min) from 50 to 700°C with a ramp rate of 10°C/min.

The acidic properties (strength and amount of acid sites) of the catalysts were determined by acid-base titration. The relative pKa values of the samples were estimated using the Gran plot analysis. Typically, 0.1 g of the sample was weighted and suspended in 10 ml of D.I. water, and then was titrated potentiometrically by continuous addition of 5 mM KOH aqueous solution.

### Catalytic evaluation

All the HDO of phenol experiments were performed in a 100 mL stainless steel batch autoclave reactor. In a typical experiment, a certain mass of phenol dissolved in a certain volume of dichloromethane was added into the reactor with a certain amount of catalysts. Before each run, the reactor was purged with hydrogen for five times, 2.0 MPa at a time, to remove air from the setup. Then hydrogen was introduced into the reactor with a certain pressure ranged from 1.0 to 4.0 MPa. The reaction system would be heated to reaction temperatures (80–200°C) within half an hour. The stirring speed and the granule size were adopted at 800 rpm and smaller than 400 mesh to ensure that the mass transfer on catalytic performances was excluded. After reactions, the reactor was put into ice water and quickly cooled down to room temperature. Subsequently, the gas in the reactor was released very slowly to a sampling gas bag that was purged in advance with H_2_ for five times. The products in gas phase were analyzed using a gas chromatography (GC) equipped with a Porapak Q packed column and a TCD. The products in liquid phase were identified and quantified by a GC-MS and a GC (equipped with an SE-30 nonpolar capillary column and a flame ionization detector, FID), and the internal standard method was applied.

Reproducibility of experiment results was carried out by repeating each run for three times at least until the results were within acceptable limits. The conversion of PhOH (X(PhOH)), yield of i (Y(i)) and the selectivity to i (S(i)) were calculated based on the following Equations. (1–3):

(1) $$\begin{array}{rcl} X\left(PhOH\right) & = & \frac{m\left(PhOH,in\right)-m\left(PhOH,out\right)}{m\left(PhOH,in\right)}\times 100\% \end{array}$$

(2) $$Y\left(i\right)=\frac{\frac{\text{m}\left(\text{i}\right)}{\text{M}\left(\text{i}\right)}\times \text{M}\left(\text{PhOH}\right)}{m\left(PhOH,in\right)}\times 100\%$$

(3) $$\begin{array}{rcl} S\left(i\right) & = & \frac{Y\left(i\right)}{X\left(PhOH\right)}\times 100\% \end{array}$$

## Results and discussion

### Characterization results

#### XRD results

**Figure 1:**
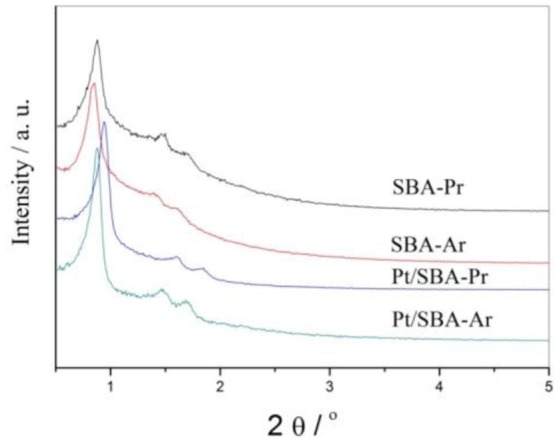
SAXRD patterns of the catalyst samples.

Figure 1 is small-angle XRD spectra of freshly prepared SBA-Pr, SBA-Ar, Pt/SBA-Pr, and Pt/SBA-Ar. Three well-resolved peaks were observed in each sample, which were indexed to the (100), (110), and (200) reflections of the hexagonal space group P6 mm (Zhao et al., 1998; Yue et al., 1999). All the diffraction patterns suggested that the SBA-15 structure was well preserved in the process of synthesis of SBA-Pr, SBA-Ar by co-condensation method and in the process of introduction of Pt by reduction-deposition method. The addition of precursors of MPTMS, CSPTMS and H_2_PtCl_6_ did not destroy the structure of the SBA-15. Figure S1 presents wide-angle XRD spectra of Pt/SBA-Pr and Pt/SBA-Ar. Only the characteristic diffraction peaks of metallic Pt, (111), (200), (220), were detected for the two samples, demonstrating that the Pt species were primarily present in metallic form after reduction by formaldehyde. According to calculation based on the Scherrer equation, the average Pt particle size of 1%Pt/SBA-Pr was 8.9 nm, while that of 1% Pt/SBA-Ar was 9.7 nm. This illustrated that Pt particles were highly dispersed on these supports.

#### DTG characterization

The differential thermogravimetric (DTG) analysis was used to determine the thermal decomposition behaviors of the tethered organic moieties in the catalyst samples, as determined by differential thermogravimetric (DTG) analysis, (shown in Figure S2). For SBA-Pr without extraction by ethanol, three peaks centered at about 110, 250, and 460°C were observed, which were resulted from the desorption of water, the decomposition of template and the decomposition of propylsulfonic acid, respectively. No weight loss peak appears in the vicinity of the 350°C (which was attributed to the decomposition of the propylthiol groups, MPTMS) (Tang et al., 2010), meaning the effective oxidation of MPTMS to propylsulfonic acid by hydrogen peroxide added during the preparation of SBA-Pr. For SBA-Pr after ethanol extraction, only weight loss peak of propyl sulfonic acid (around 470°C) was observed on DTG curves, indicating complete removal of the template P123 by ethanol extraction method. The results of DTGclearly showed that the propylsulfonic acid groups in the catalysts did not occur decomposition while using the catalyst under 350°C owing to the higher thermal stability of tethered propylsulfonic acid.

#### ICP-MS and CO chemisorptions results

The properties of the metal sites of two catalysts, including metal loading, and dispersion, were characterized by ICP-MS and CO chemisorption techniques. The results are listed in Table 1. ICP results showed that the real loading of Pt was smaller than the theoretical loading of 1 wt.%, demonstrating loss of metal during the preparation process. When Pt was loaded on SBA-Pr, the highest load efficiency of 84% and the highest dispersion (D_Pt_) of 53% were obtained. The metal particle sizes (d_Pt_) showed in Table 1 were calculated from the CO chemisorptions results according literature (Wang and Yeh, 2001). All the metal particles of Pt were highly dispersed over the SBA-Pr and the SBA-Ar with d_Pt_ < 3.0 nm. Noticeably, the d_Pt_ was as small as 2.1 nm on the Pt/SBA-Pr. Evidently, the particle sizes calculated from chemisorptions was quite smaller than the aforementioned data derived from XRD. It is well known that the XRD can only be sensitive the big crystallines but not the highly dispersed particles.

**Table 1:** Metal sites properties of the catalysts.

**Catalysts**	**Metal content/wt.%**	**D_Pt_/%**	**d_Pt_/nm**
Pt/SBA-Pr	0.84	53	2.1
Pt/SBA-Ar	0.60	41	2.7

#### TEM characterization results

**Figure 2:**
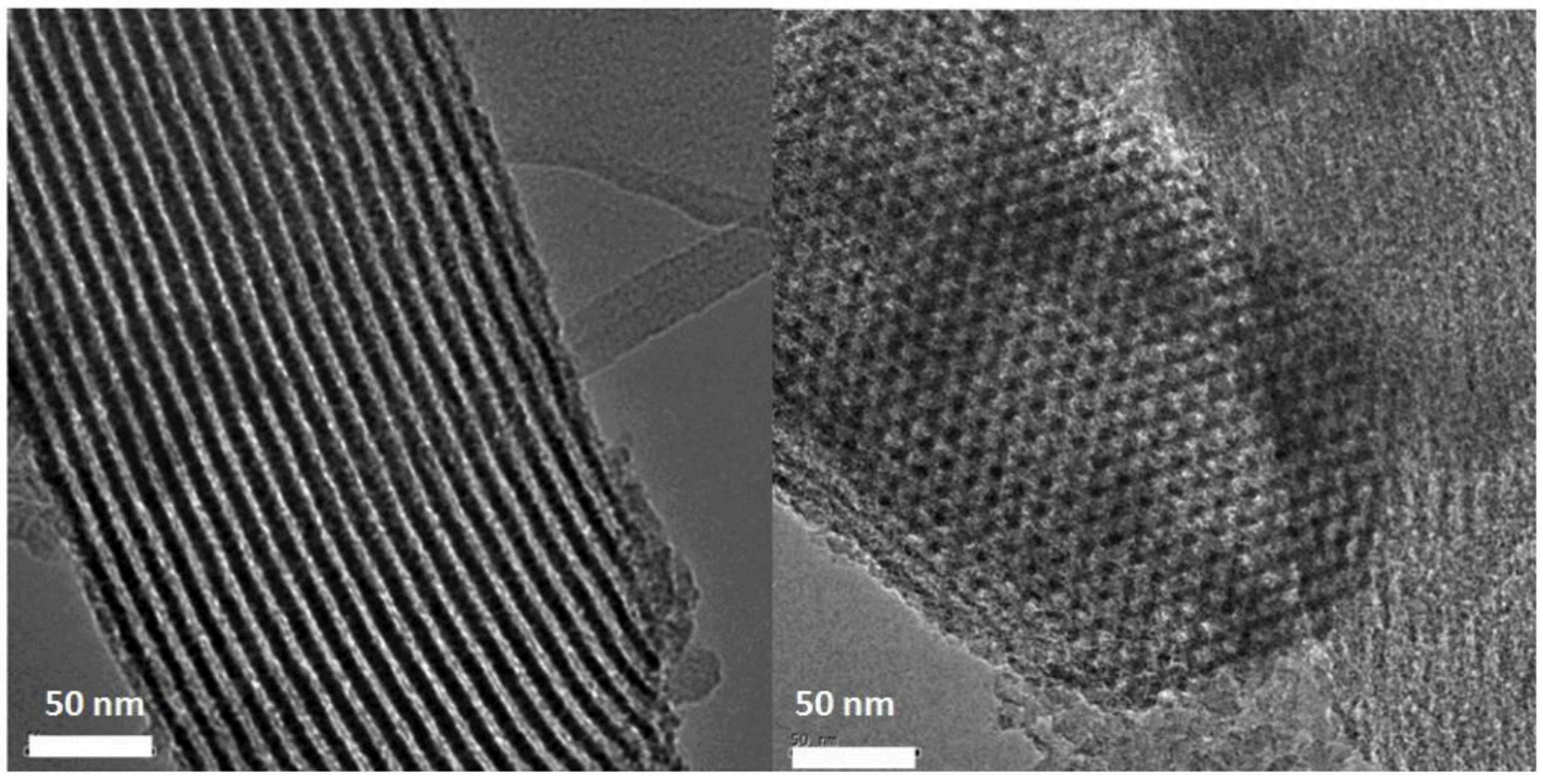
TEM images of sample of SBA-Pr.

**Figure 3:**
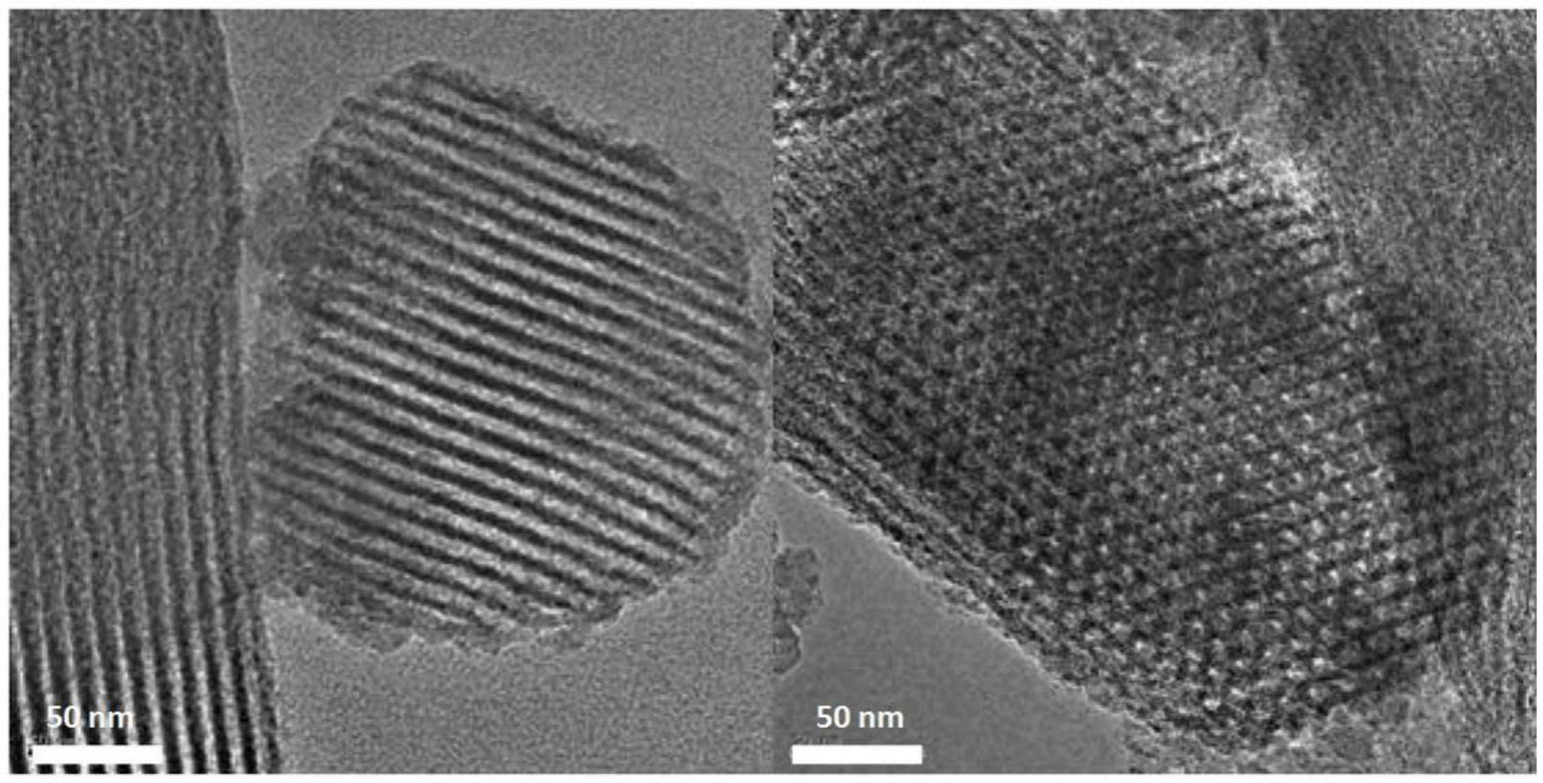
TEM images of sample of SBA-Ar.

**Figure 4:**
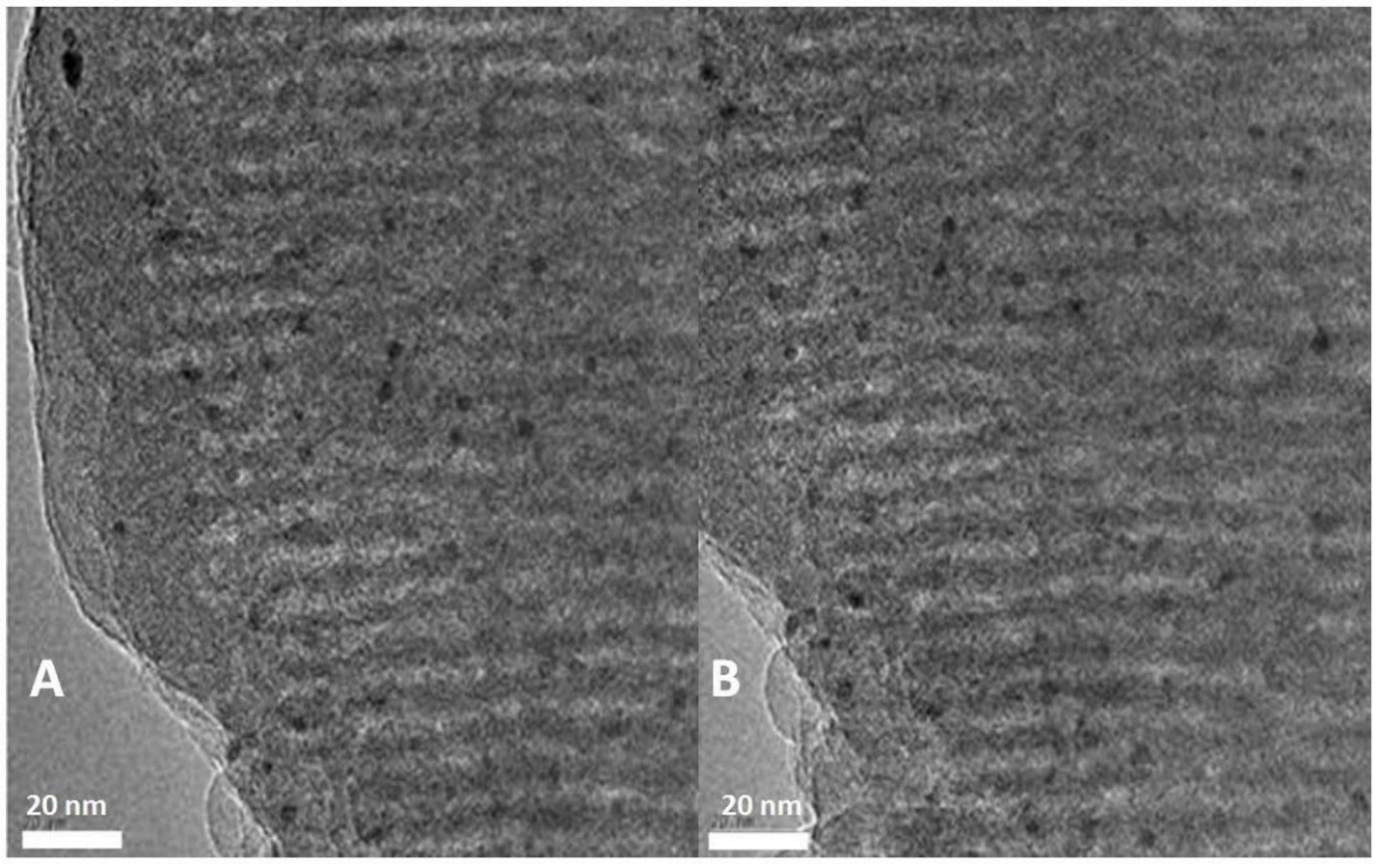
TEM images of 1%Pt/SBA-Pr **(A)** and 1%Pt/SBA-Ar **(B)**.

TEM is a useful technique to characterize the structure of mesoporous materials. From Figures 2, 3, it can be seen, the organic sulfonic acid functionalized SBA-15 materials have regular one-dimensional pore structure preserving the characteristic pore structure of SBA-15. Figure 4 shows that after loading of Pt the mesoporous structure of the carriers were still well preserved and the Pt particles were dispersed well on the supports. It could be observed that a lot of Pt particles were as small as <2 nm located in the mesopores.

#### N_2_ adsorption–desorption characterization results

The N_2_ adsorption-desorption isotherms of the samples of SBA-Pr, SBA-Ar, Pt/SBA-Pr, and Pt/SBA-Ar are shown in Figure S3. The observed isotherms of the four samples were obviously classified as IV type isotherms, which was consistent with the mesoporous structure directed by non–ionic surfactant (Buchmeiser, 2003). All the isotherms had Type-H 1 hysteresis loops, and the capillary condensation occured at higher relative pressures (0.7–0.8). This illustrated the synthesized mesoporous materials possessing regular pore arrangements and narrow pore size distributions (Schmidt et al., 1995). The nitrogen sorption isotherms also revealed that no partial pore blocking has occurred upon incorporation of platinum, as the adsorption and desorption branches were almost parallel and exhibited narrow hysteresis. After loading of platinum the pore diameter, pore volume, and surface area of SBA-15 were waned showing in Table S1. After functionalization with organosulfonic acid and loading of Pt, the specific surface area and pore size of the samples were decreased compared to SBA-15, but still remained at a high level. These data also showed that the organic and inorganic functional groups did not cause remarkable clogging of pores. The structure of SBA-15 remained stable during the functionalization.

### Catalytic testing results

#### Pt based organic-inorganic hybrid materials catalyzed HDO reaction of phenol

The catalytic results of phenol HDO catalyzed by bifunctional Pt/SBA-Pr, as well as the relevant control experiment results are presented in Table 2. From Entry 1, a good result of phenol HDO reaction was achieved over the bifunctional catalyst of 1%Pt/SBA-Pr with high conversion of phenol (X(PhOH) = 94.1%) and high selectivity to target product of cyclohexane (S(C_6_H_12_) = 98.6%). From Entry 3 and 5, it could be seen that when there is no catalyst or SBA-Pr is used alone phenol did not convert at all. Surprisingly, in Entry 2, over the catalysts of 1% Pt/SBA-15 the product of cyclohexane was also formed and the selectivity was as high as 63.8%. In entry 4, 1%Pt/SBA-15 and SBA-Pr was physically mixed to form bifunctional catalyst. It was found that both of X(PhOH) and S(C_6_H_12_) were significantly improved over the physically mixed catalyst compared to the 1%Pt/SBA-15 catalyst (entry 2). From above results, it could be considered that the metal sites of catalysts alone catalyze the HDO of phenol with the aid of negligible acidity sites of silanol groups on SBA-15 support, but the stronger acid sites would greatly enhance the HDO reaction activity of the catalyst. Compared to the physical mixed bifunctional catalyst (entry 4), the composite bifunctional catalyst (entry 1) owed the highest conversion of phenol and the best selectivity to cyclohexane. The above results exhibited that there was a synergistic effect between metal sites and acid sites for HDO reaction of phenol. Huang and Baiker group also found that the proper acid sites properties could significantly enhance the catalytic performance for the reaction of hydrogenation of acetophenone on the Pt/[Al]MCM-41 (Wang Z. et al., 2015).

**Table 2:** Catalytic performance for HDO of phenol.

**Entry**	**Catalysts**	**X(PhOH)/%**	**Y(C_6_H_12_)/%**	**S(C_6_H_12_)/%**
1	1%Pt/SBA-Pr	94.1	92.8	98.6
2	1%Pt/SBA-15	44.8	28.6	63.8
3	SBA-Pr	0	0	–
4	1%Pt/SBA-15+SBA-Pr	74.5	63.3	84.9
5	Catalyst-free	0	0	–

*Reaction conditions: T_R_, 200°C; P_H2_, 4.00 MPa; t_R_, 8 h, 800 rpm, 0.0125 mol PhOH in 25.0 mL CH_2_Cl_2_, 0.9756 g catalyst (0.05 mmol Pt, except for entry 3 and 5)*.

#### Effect of supports on catalyst performances

Pt was supported on SBA-Pr and SBA-Ar to form bifunctional catalysts of 1%Pt/SBA-Pr and 1%Pt/SBA-Ar. Their catalytic results for HDO of phenol are listed in Table 3. 1%Pt/SBA-Pr exhibited higher conversion of phenol (94.1%) and higher selectivity to cyclohexane (98.6%), compared to SBA-Ar supported Pt catalyst with X(PhOH) = 35.0% and S(C_6_H_12_) = 67.4%. According to the characterization results (Table 1), though the two catalysts had the same nominal Pt loading, they actually had different Pt content. In order to exclude the influence of the metal content on the results, the TON calculated based on the target product of cyclohexane are also shown in Table 3. Pt/SBA-Pr shows higher TON (103.3 mol cyclohexane/mol Pt) in comparison with Pt/SBA-Ar (14.5 mol cyclohexane/mol Pt). Since the impact of metal sites on HDO reaction was excluded, only the acidities of the supports affected the HDO reaction.

**Table 3:** Pt supported on different carriers for HDO of phenol.

**Support**	**X(PhOH)/%**	**Y(C_6_H_12_)/%**	**S(C_6_H_12_)/%**	**TON(480 min)**
SBA-Pr	94.1	92.8	98.6	103.3
SBA-Ar	35.0	23.6	67.4	14.5

*Reaction conditions: T_R_, 200°C; P_H2_, 4.0 MPa; t_R_, b8 h, 800 rpm; t_R_, 4 h, 0.0125 mol PhOH in 25.0 mL CH_2_Cl_2_, 0.9756 g catalyst (n_Pt_:n_PhOH_ = 1:250)*.

The acidities of the catalysts, including the amount and strength of the acid sites, were characterized by means of acid-base titration. The results are shown in Table 4. In spite of the same nominal loading, the real amount of acid sites of SBA-Ar was smaller than that of SBA-15-Ar. This might be related to the fact that the molecule of arenesulfonic acid had bigger size and rigidity compared to propylsulfonic acid, which was not conducive to effective loading of arenesulfonic acid. The data also showed that the pKa value of SBA-Ar was smaller than that of SBA-Ar, i.e., SBA-Ar exhibited stronger acid sites. According to Shanks (Mbaraka and Shanks, 2006), the arenesulfonic acid-functionalized samples had lower pKa values as the sulfonate ions in the phenyl group were more stablethan that in aliphatic carbon chain of propylsulfonic acid groups after deprotonation.

**Table 4:** Acidities of the catalysts and the supports.

**Catalysts**	**Acid Sites/(μmol/g)**	**pKa**
SBA-Pr	810	3.72
Pt/SBA-Pr	790	3.61
SBA-Ar	560	3.26
Pt/SBA-Ar	470	3.18

Pt/SBA-Pr had more acid sites but lower acid strength compared to Pt/SBA-Ar (Table 4). The catalytic activity of Pt/SBA-Pr was higher than that of Pt/SBA-Ar (Table 3). Therefore, it was concluded that the acid sites amount of the supports may be a dominant factor to affect the activity of a bifunctional catalyst. The more the acid sites was, the higher activity of the HDO reaction was. In addition, from Table S1, the pore volume of Pt/SBA-Pr and Pt/SBA-Ar is 0.91 and 0.78 cm^3^/g, respectively. Obviously, from perspective of mass diffusion the former was in favor of the reaction.

#### Screening of solvents

Solvent always plays an important role in a liquid phase reaction. Table 5 presents the results of phenol hydrodeoxygenation in different solvents. The catalytic activity of 1%Pt/SBA-Pr was the lowest (X(PhOH)~5.0%) as the solvent of water with high polarity was used, while the solvent of dicloromethane with low polarity was adopted both the X(PhOH) and the S(C_6_H_12_) reaches a maximum with 63.0 and 95.9%, respectively. From the above results, solvents had significant effects for HDO of phenol, the catalytic activities increased as the polarity of a solvent lowered. The solvent of dichloromethane with the lowest polarity was the best reaction solvent among the solvents tested, including H_2_O, EtOH, dioxane, and dicloromethane solvents. So the optimization work was carried out in the solvent of dichloromethane.

**Table 5:** HDO of phenol over 1%Pt/SBA-Pr in different solvents.

**Solvent**	**X(PhOH)/%**	**Y(C_6_H_12_)/%**	**S(C_6_H_12_)/%**
H_2_O	5.0	3.7	74.0
EtOH	6.7	3.5	52.2
Dioxane	25.0	19.4	77.6
CH_2_Cl_2_	63.0	60.4	95.9

*Reaction conditions: T_R_, 200°C; P_H2_, 3.0 MPa, 800 rpm; t_R_, 4 h, 0.0250 mol PhOH in 50.0 mL solvent, 0.4878 g 1%Pt/SBA-Pr (n_Pt_:n_PhOH_ = 1:1000)*.

#### Optimization of reaction conditions

**Figure 5:**
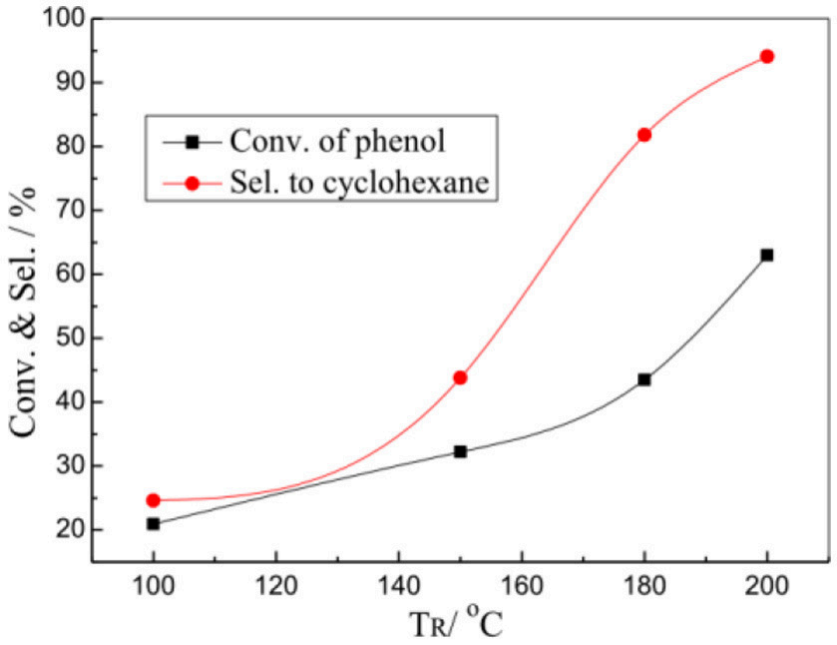
Effect of T_R_ on the HDO of phenol over 1%Pt/SBA-Pr. Reaction conditions: P_H2_, 3.00 MPa; 800 rpm; t_R_, 4 h, 0.0250 mol PhOH in 50.0 mL CH_2_Cl_2_, 0.4878 g 1%Pt/SBA-Pr (n_Pt_:n_PhOH_ = 1:1,000).

Figure 5 shows the effects of reaction temperature (TR) on the HDO reaction of phenol over the 1%Pt/SBA-Pr catalyst. Both conversion of phenol and selectivity to cyclohexane increased with the T_R_ increasing. In the evaluation range of temperature, at 200°C the X(PhOH) and the S(C_6_H_12_) reaches maximum, 63.0 and 95.5%, respectively. The main byproduct was cyclohexanol.

**Figure 6:**
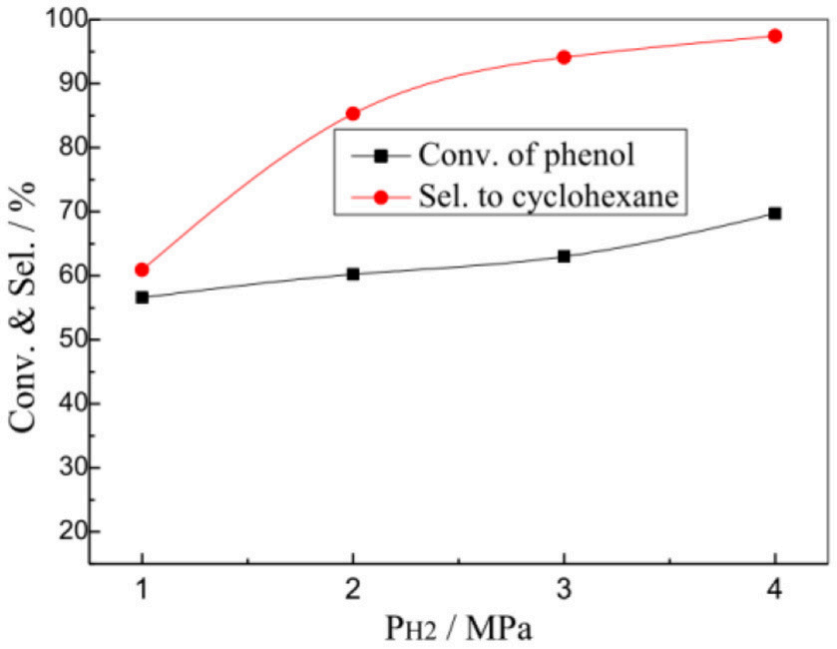
Effect of P_H2_ on the HDO of phenol over 1%Pt/SBA-Pr. Reaction conditions: T_R_, 200°C, 800 rpm; t_R_, 4 h, 0.0250 mol PhOH in 50.0 mL CH_2_Cl_2_, 0.4878 g 1%Pt/SBA-Pr (n_Pt_:n_PhOH_) = 1:1000).

Figure 6 presents the effects of hydrogen pressure (P_H2_) on the HDO reaction of phenol over the 1% Pt/SBA-Pr catalyst. In the evaluation range the initial hydrogen pressure had little effect on the conversion of phenol, it mainly affected the selectivity to the desired product cyclohexane. The X(PhOH) increased from 56.6 to 69.7% while increasing the P_H2_ from 1.0 MPa to 4.0 MPa. However, the S(C_6_H_12_) increased remarkably from 60.9 to 97.4%. The higher the initial hydrogen pressure was, the larger the concentration of adsorbed active hydrogen on the catalyst surface was, and thus the more conducive to the generation of the target product of cyclohexane.

**Figure 7:**
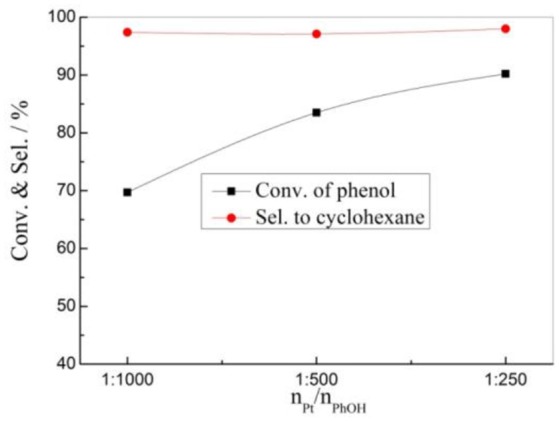
Effect of n_Pt_/n_PhOH_ on the HDO of phenol over 1%Pt/SBA-Pr. Reaction conditions: P_H2_, 4.00 MPa; T_R_, 200°C; 800 rpm, t_R_, 4 h.

The effects of the catalyst amount on the HDO reaction of phenol was also investigated over the 1% Pt/SBA-Pr catalyst (Figure 7). At the certain reaction condition (P_H2_ = 4.00 MPa, T_R_ = 200°C, 800 rpm, t_R_ = 4 h), the conversion of phenol increased with the n_Pt_/n_PhOH_ increasing from 1:1000 to 1:250, while the selectivity to the target product of cyclohexane remains almost unchanged, meaning that the selectivity to cyclohexane was independent on the amount of Pt metal sites under this reaction condition.

**Figure 8:**
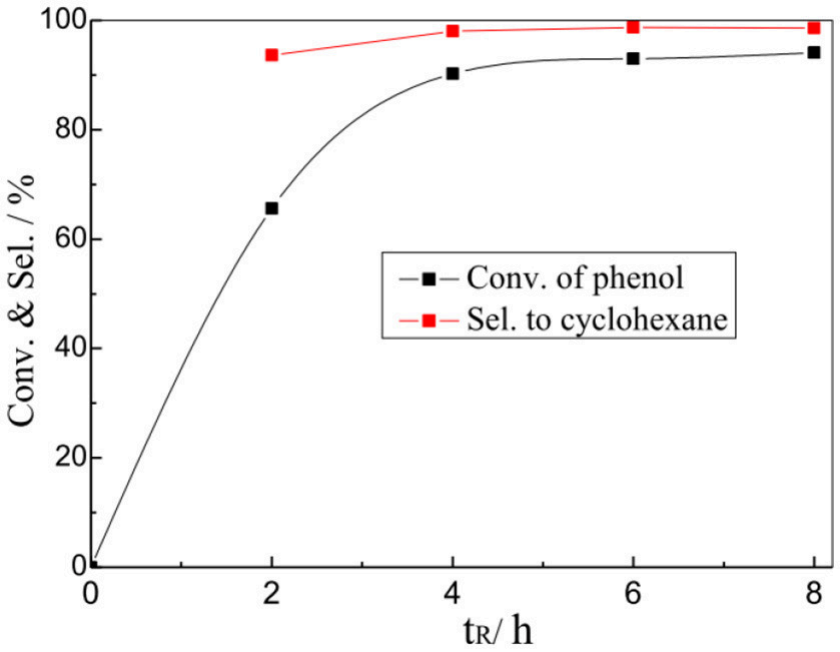
Effect of t_R_ on the HDO of phenol over 1%Pt/SBA-Pr. Reaction conditions: P_H2_, 4.0 MPa, TR, 200°C; 800 rpm, 0.0125 mol PhOH in 25.0 mL CH_2_Cl_2_, 0.9756 g 1%Pt/SBA-Pr (n_Pt_:n_PhOH_ = 1:250).

The effect of reaction time on the catalytic performance of 1%Pt/SBA-Pr was also investigated. Most of the phenol was converted within the first 2 h (Figure 8). When increasing the reaction time from 4 h to 8 h, the conversion of phenol slightly increased from 90.2 to 94.1%, with the selectivity to cyclohexane almost unchanged.

By screening the reaction temperature, the initial hydrogen pressure, reaction time and the relative content of the catalyst and the reaction solvent, the optimal reaction condition was obtained: T_R_ = 200°C, P_H2_ = 3.00 MPa, 800 rpm, t_R_ = 4 h, 0.0125 mol PhOH in 25.0 mL CH_2_Cl_2_, 0.9756 g 1%Pt/SBA-Pr (n_Pt_:n_PhOH_) = 1:250. Under these conditions, the conversion of phenol was 94.1%, and the selectivity to desired product of cyclohexane reached to 98.6%.

#### Possible reaction mechanism

**Scheme 1:**
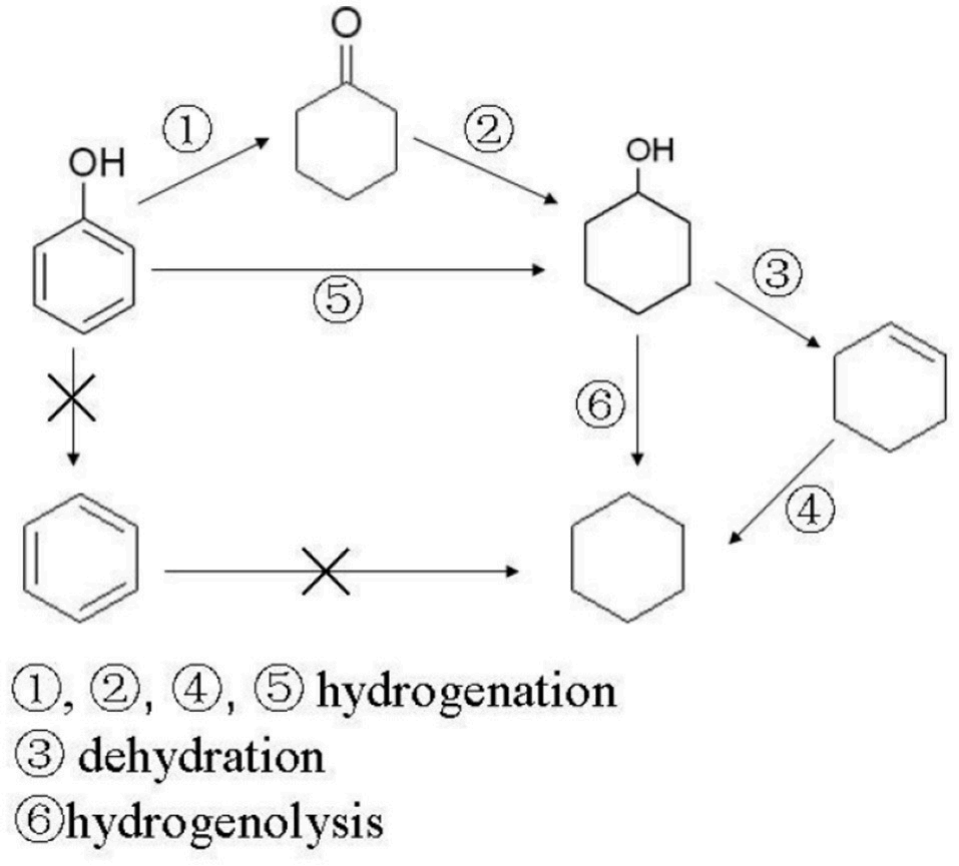
Proposed reaction mechanism for HDO of phenol.

Based on the experimental results a possible reaction mechanism for HDO of phenol was proposed as Scheme 1. Experimental results of Kou et al (Yan et al., 2010; Zhao et al., 2010) showed that cyclohexanol can exist stably under acid catalysts below 180°C. Once the temperature was above 180°C under acid catalysts it dehydrated rapidly to produce cyclohexene. But in our reaction conditions, cyclohexene was never detected as, cyclohexene might be easily hydrogenated to cyclohexane in the reaction system. At lower reaction temperatures (below 180°C), or when there are no acidic sites on the catalyst, cyclohexanol cannot dehydrate to form cyclohexene, so hydrogenation reaction is conducted by path 172173 or 176 to generate cyclohexanol, hydrogenolysis of which takes place to form cyclohexane follow the path of 177. Different from the literatures (Hong et al., 2010; Zhu et al., 2011) performing with gas phase reactants in fixed bed reactor, aromatic hydrocarbons, which were produced from hydrogenolysis (direct deoxygenation), could not be found in this work. The difference might result from the different reaction conditions and catalyst systems. Therefore, the reaction pathways might be proposed as the Scheme 1.

## Conclusions

In this work, organic-inorganic hybrid materials of SBA-Pr and SBA-Ar were synthesized and loaded with Pt to form bifunctional catalysts. These bifunctional catalysts had good catalytic activities for HDO of phenol to produce cyclohexane, a model reaction of biofuel upgrading. Under optimal reaction conditions, the conversion of phenol was 94.1%, with 98.6% selectivity to the target product of cyclohexane. The metal sites and the acid sites on bifunctional catalysts displayed significant synergistic effect for the HDO of PhOH. The bifunctional catalysts using in this work may be potentially applied to the upgrading of bio-fuel derived from lignin or biomass.

## Author contributions

LM was in charge of the writting and designing the experiments. WY and HC performed the experiments. XZ and HL were consultants to the project.

### Conflict of interest statement

The authors declare that the research was conducted in the absence of any commercial or financial relationships that could be construed as a potential conflict of interest.
