# Supplementary material for: Hydrodeoxygenation of Bio-Derived Phenol to Cyclohexane Fuel Catalyzed by Bifunctional Mesoporous Organic–Inorganic Hybrids
Source: Front Chem. 2018 Jun 14;6:216. doi: 10.3389/fchem.2018.00216 (PMC6010534; doi:10.3389/fchem.2018.00216)
Supplement: Supplementary file 1 [file Presentation_1.PPTX]

## Slide 1
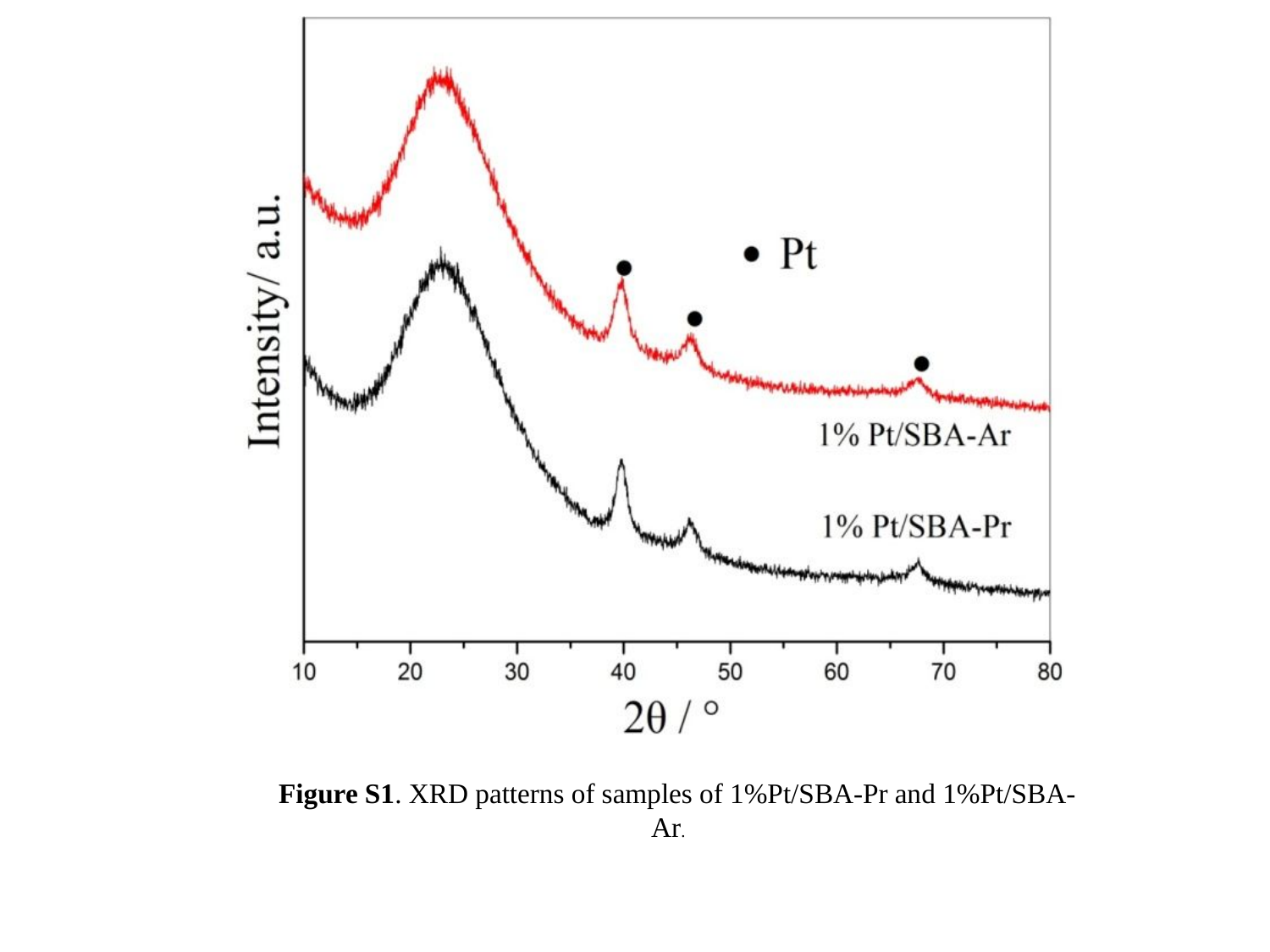

Figure S1. XRD patterns of samples of 1%Pt/SBA-Pr and 1%Pt/SBA-Ar.

## Slide 2
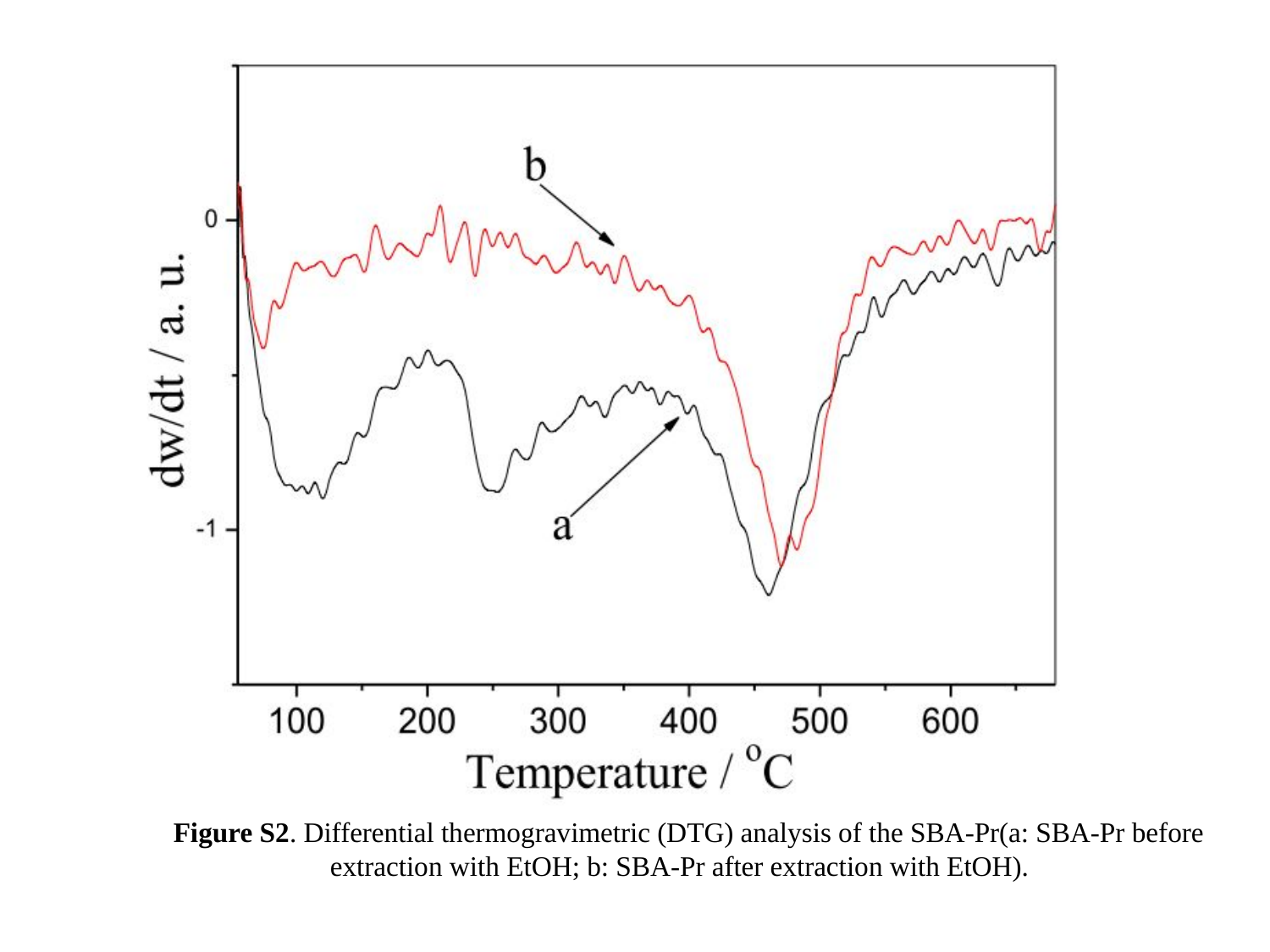

Figure S2. Differential thermogravimetric (DTG) analysis of the SBA-Pr(a: SBA-Pr before extraction with EtOH; b: SBA-Pr after extraction with EtOH).

## Slide 3
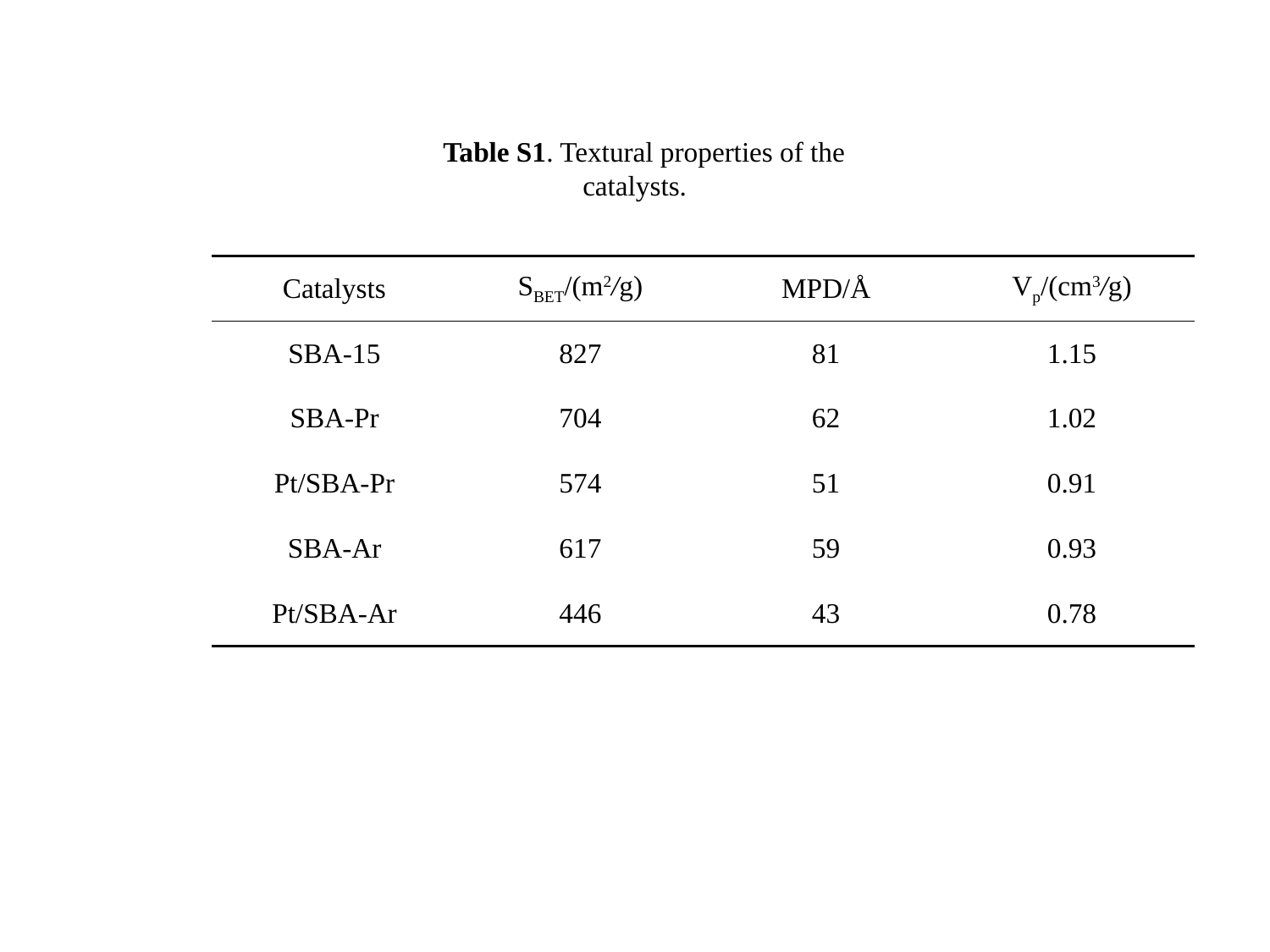

Table S1. Textural properties of the catalysts.
| Catalysts | SBET/(m2/g) | MPD/Å | Vp/(cm3/g) |
| --- | --- | --- | --- |
| SBA-15 | 827 | 81 | 1.15 |
| SBA-Pr | 704 | 62 | 1.02 |
| Pt/SBA-Pr | 574 | 51 | 0.91 |
| SBA-Ar | 617 | 59 | 0.93 |
| Pt/SBA-Ar | 446 | 43 | 0.78 |

## Slide 4
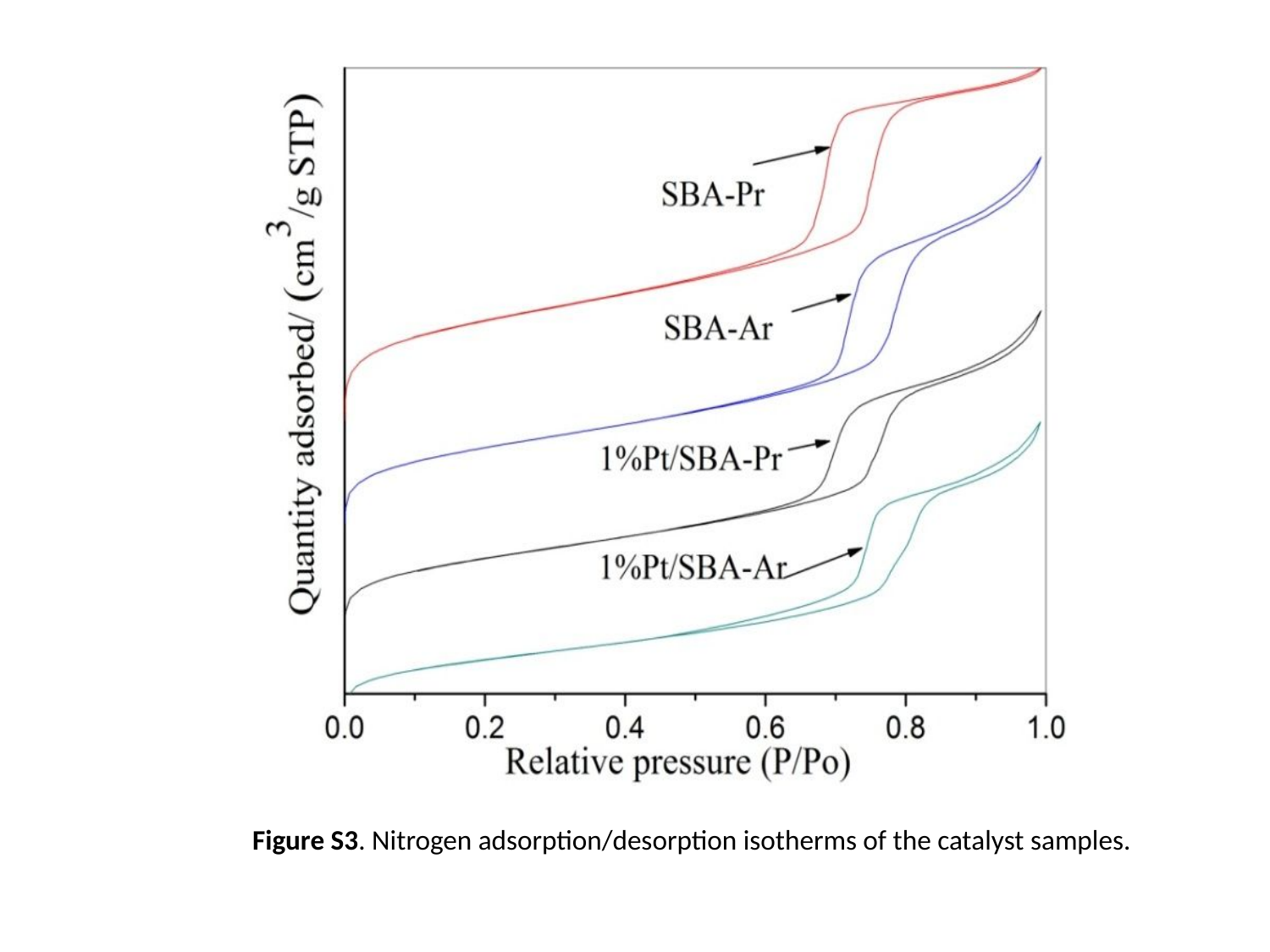

Figure S3. Nitrogen adsorption/desorption isotherms of the catalyst samples.
